# Laparoscopic Management of a Recurrent, Inguinal Hernia Containing Urinary Bladder: A Case Report and Literature Review

**DOI:** 10.1155/carm/2076137

**Published:** 2025-07-08

**Authors:** Abdolreza Mohammadi, Pedram Golmohammadi, Alireza Pakdel, Seyed Mohammad Kazem Aghamir

**Affiliations:** Urology Research Center, Tehran University of Medical Sciences, Tehran, Iran

**Keywords:** incisional, inguinal hernia, laparoscopy, urinary bladder

## Abstract

**Background:** Inguinal hernia is one of the most common causes of surgeries around the world; however, the herniation of the urinary bladder as a recurrence in this region is rare. It can have urinary symptoms with pain and protrusion in the inguinoscrotal region.

**Case Presentation:** We report the case of a 65-year-old Iranian male patient who presented with obstructive urinary symptoms and a reducible protrusion in the right inguinoscrotal region. The patient underwent a successful repairment operation in a laparoscopic manner without any intra- or postoperative complications. Also, here, we review the risk factors, symptoms, and paraclinics that should be noted regarding the inguinal hernia with visceral organ protrusion.

**Conclusion:** Clinicians must be aware of different symptoms of herniated organs in the inguinal region to avoid unwanted complications.

## 1. Introduction

Inguinal hernia is a challenging problem due to its recurrence rate after abdominal surgeries. However, the inguinal urinary bladder hernia as a recurrence is rare in prevalence and includes small percentage of all inguinal hernias. The urinary bladder inguinal hernia usually shows as a different-sized protrusion in the inguinal or scrotal region. It can be spontaneously reducible, particularly after voiding or not. Some patients have urinary symptoms, including incomplete voiding, abdominal pain, and even obstructive urinary symptoms [1, 2]. It also may have no urinary symptoms, just as a usual inguinal hernia.

Herein, we aim to introduce a case of recurrent inguinal hernia containing urinary bladder that underwent a laparoscopic repair with a subsequent literature review.

## 2. Ethics Statement

This case is reported based on CARE guidelines were developed by an international group of experts to support an increase in the accuracy, transparency, and usefulness of case reports [3]. The patient agreed to report his case by signing the written informed consent.

## 3. Case Presentation

A 65-year-old man with incomplete voiding and frequency complaints from 6 months ago was referred to our urology clinic. He also mentioned straining without hesitancy during voiding. The patient informed us that he had an open surgery for an inguinal hernia about 3 years ago in another hospital. He did not mention any history of urinary tract disease or related treatments.

In physical examination, we found a 30 ∗ 50 ∗ 20 mm protrusion in a right inguinal area with about 6 cm of skin scar at the same place, which was reducible by hand and had no pain or tenderness. The patient did not notice a change in its size during the urination. The scrotum and both testicle sizes were normal and symmetric. The digital rectal examination was done with normal findings as well as other physical exams.

**Figure 1:**
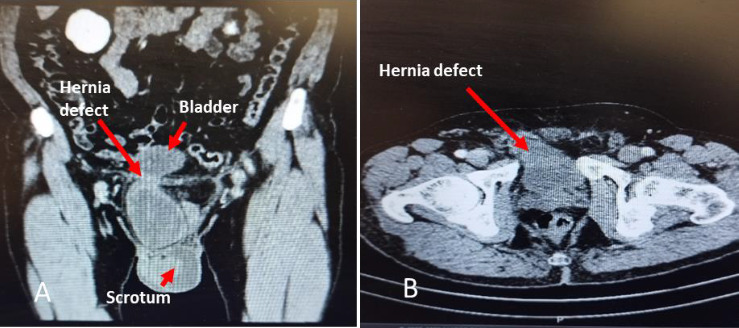
(A) Spiral abdominal and pelvic with IV contrast CT scan in the coronal view. The vertical view in (B) illustrates bladder herniation defect, indicated by red arrows.

He accompanied a report of urinary tract and superficial probe ultrasonography (7.5 kHz). He noted a protrusion of the urinary bladder with about 30 cc volume and a small piece of omentum fat in the left inguinal region. Also, the prostate size was reported to be about 30 cc, and other findings were normal. The lab tests, which included complete blood cell, serum creatinine, urine analysis, and culture, were normal. Figures 1(A), 1(B) depict spiral abdominal and pelvic CT scan with intravenous contrast injection in which the urinary bladder is protruded through the right inguinal hernia defect to the inguinal canal.

**Figure 2:**
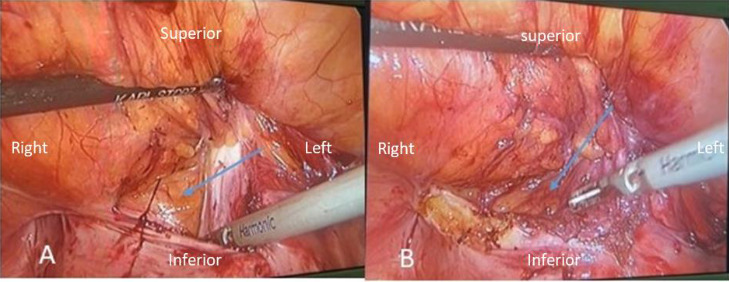
(A) and (B) depict the laparoscopic surgery imaging of a urinary bladder hernia. Arrows point to perivesical fat in the posterior wall of the right inguinal canal.

After consulting with the patient, a laparoscopic approach was chosen for the management of his inguinal hernia. His previous surgery document just noted a peritoneal sac in the inguinal canal and a repairment without mesh insertion. At first the urethral Foley catheter was inserted. In the transabdominal preperitoneal (TAPP) technique, the three ports were placed at the umbilicus and the area of the mid-clavicular line at the level of the umbilicus on the left and right side of the abdomen. We found a protrusion of the posterior wall of the right inguinal canal through Hesselbach's triangle, and the incisional inguinal bladder herniation was confirmed. This hernia was successfully repaired with careful exploration and division of the adhesive peripheral tissues in the usual laparoscopic manner, and a dual mesh was inserted.  Figure 2 presents the laparoscopic view of bladder herniation and a last view of repairment.

The total surgery time was 45 min. Paracetamol was used as a painkiller with a dose of 10 mg/kg/6 h intravenously during the first day then he was discharged with oral painkillers for 1 week but he then informed us that he had no need them after 2 days. After 3 months follow-up; there were no complications, and he was satisfied with his treatment.

## 4. Discussion

The inguinal hernia repair is one of the most common surgeries around the world. According to a high number of patients and its common side effects, such as chronic pain and recurrence after surgery, various treatments have been developed. Previous studies have shown that recurrence of the inguinal hernia usually occurs 1–5 years after the first surgery, and in our case, it occurred in the third year [3]. An inguinal hernia can contain an omental fat-intestine or colon loop. In rare instances, it may even involve the bladder or ureter, leading to symptoms like incomplete voiding and frequent urination. In radiologic findings, it may also observe hydroureteronephrosis. Also, in some cases, it may be asymptomatic or not diagnosed preoperatively, so the surgeon must be vigilant regarding urinary bladder herniation [4]. Most bladder hernias occur in the inguinal and femoral regions. Its occurrence in the form of recurrent hernia is rare. It is mainly reported after urological surgeries such as stress urinary incontinence and retropubic prostatectomy, but it is rare after abdominal surgeries such as inguinal hernia repair [5]. The inguinal hernia has genetic and acquired causes, usually in children and adults [6]. The risk factors include family history, previous contra-lateral hernia, male gender, advanced age, abnormal collagen metabolism, prostatectomy, and low body mass index. In addition, the perioperative risk factors for recurrence include poor surgical techniques, low surgical volumes, surgical inexperience, and local anesthesia. The patient's properties allow for a successful laparoscopic repairment using the TAPP approach and dual mesh insertion, all done under general anesthesia. To prevent a second recurrence, it seems to be a low-risk management option [7]. Although most surgeons only use clinical symptoms and physical examination to diagnose the inguinal hernia, due to the possibility of different organs existing in the inguinal hernia sac, the role of imaging, especially ultrasonography, cannot be ignored before the surgery. Voiding cystourethrography (VCUG) is mentioned as a diagnostic imaging in bladder hernia, which can help find underlying causes of hernias such as lower urinary tract strictures and also in bladder hernias that are symptomatic only during voiding. Spiral abdominopelvic CT scan with IV contrast and delayed phase in those with a history of previous surgery in the inguinal or abdominopelvic area can help differentiate bladder hernia from the ureter, colon, and other organs herniation, as well as in people with the age more than 50-year-old to rule out possible malignancies that may be related to the occurrence of this hernia [8]. We performed a CT scan with the mentioned protocol, so we found more anatomical information and ruled out the other differential diagnosis, as shown in Figure 1.

The role of MRI, especially in cases of hernia recurrence and in differentiating the cause of inguinal pain after surgery, has been evident [9]. Also, the spiral abdominopelvic CT scan during the Valsalva maneuver has shown high specificity and accuracy for the diagnosis of inguinal hernia [10]. One of the common complications after this hernia repairment is urinary retention. Male gender, aging 65 years or older, anticholinergic medication, history of urinary retention, constipation, out-of-hours surgery, involvement of urinary bladder within the hernia, temporary intraoperative urethral catheterization, and increasing operative duration are the risk factors of this problem. In our presented case, for urinary bladder decompression and considering that the patient was at high risk of urinary retention due to hernia surgery, a 16 French-sized Foley catheter was inserted [11]. We did not find any nonabsorbable thread or evidence of previous surgical mesh insertion in the surgical site; therefore, according to previous studies, the lack of mesh insertion can be one of the potential reasons for the recurrence of this hernia [12]. Some studies have been conducted to compare laparoscopic and open methods for repairing inguinal hernia, which show that the complications of the two methods are not significantly different. However, the postoperative pain was reported less in the laparoscopic method [13, 14]. Another study has shown that the laparoscopic extraperitoneal (TEP) groin hernia repair improves testicular functions, sexual functions, and quality of life in comparison with transabdominal preperitoneal surgery [15] Recently, a three-arm study that compared open mesh hernioplasty (OMH), TEP, and TAPP repair of groin hernia in the result there was a significant increase of antisperm antibody level in OMH group as compared to TAPP and TEP, but this level was in the normal range in all three groups and there was any significant difference between groups. Also, it is noteworthy that laparoscopic and robotic procedures are performed in developed countries. However, those can be good options for selected patients and seem dependent on patient counseling and surgeons' expertise [16, 17]. The other advantages of laparoscopic surgery are decreased incidence of complications, less pain, lower need for analgesics, faster recovery, early mobilization, and eventually, the feasibility of these minimally invasive techniques (laparoscopic and robotic) as definitive surgical repair approaches [18].

## 5. Conclusion

Today, it is common to diagnose inguinal hernia just by clinical symptoms and examination but to avoid misdiagnosis of various internal organs that may exist in the inguinal region asking for urinary symptoms and if it was positive or there were other unusual findings or in repeated hernia, some paraclinics especially imaging modalities can be helpful. Also, managing these situations in a laparoscopic manner can be a good option in selected patients.

## Data Availability

The data that support the findings of this study are available on request from the corresponding author. The data are not publicly available due to privacy or ethical restrictions.

## References

[1] Chio U.-C., Liu Y.-L., Siow T.-F., Chen K.-H. (2023). Endoscopic Totally Extraperitoneal Repair of Combined Gibson Incisional and Inguinal Hernia in a Kidney Transplant Recipient. *Asian Journal of Surgery*.

[2] McLain S., Cecire J., Mekisic A. (2023). Incarcerated Inguinal Hernia Containing Urinary Bladder and Bladder Calculi. *Journal of Surgical Case Reports*.

[3] Köckerling F., Koch A., Lorenz R., Schug-Pass C., Stechemesser B., Reinpold W. (2015). How Long Do We Need to Follow-Up Our Hernia Patients to Find the Real Recurrence Rate?. *Frontiers in surgery*.

[4] Morrison Z., Kashyap S., Nirujogi V. L. (2022). Adult Inguinal Hernia. *StatPearls*.

[5] Sasaki S., Miura E., Nakayama H., Watanabe T. (2014). An Incisional Bladder Hernia Following Appendectomy: Report of a Case. *Surgery Today*.

[6] Hammoud M., Gerken J. (2024). Inguinal Hernia. *StatPearls*.

[7] The HerniaSurge Group (2018). International Guidelines for Groin Hernia Management. *Hernia*.

[8] Ganesan G., Ramachandran R., Raji V. B. R., Nandhakumar S., Rangasami R., Sai P. M. V. (2023). A Radiological Review of the Unusual Contents of Inguinal Region. *Indian Journal of Radiology and Imaging*.

[9] Rosbach N., Wenger-Alakmeh K., Lahrsow M. (2022). The Role of Dynamic Magnetic Resonance Imaging in Exclusion of Inguinal Hernia in Patients Suffering From Indefinitive Groin Pain. *Hernia*.

[10] Ghafoor S., Tognella A., Stocker D. (2023). Diagnostic Performance of CT With Valsalva Maneuver for the Diagnosis and Characterization of Inguinal Hernias. *Hernia*.

[11] Croghan S. M., Mohan H. M., Breen K. J. (2023). Global Incidence and Risk Factors Associated With Postoperative Urinary Retention Following Elective Inguinal Hernia Repair: The Retention of Urine After Inguinal Hernia Elective Repair (RETAINER I) Study. *JAMA Surgery*.

[12] Lockhart K., Dunn D., Teo S. (2018). Mesh versus Non‐Mesh for Inguinal and Femoral Hernia Repair. *Cochrane Database of Systematic Reviews*.

[13] Sekhon Inderjit Singh H. K., Massey L. H., Arulampalam T., Motson R. W., Pawa N. (2022). Chronic Groin Pain Following Inguinal Hernia Repair in the Laparoscopic Era: Systematic Review and Meta-Analysis. *The American Journal of Surgery*.

[14] Zhu X., Liu Z., Shen J., Liu J., Tang R. (2023). Comparison of Open and Laparoscopic Inguinal-Hernia Repair in Octogenarians. *Asian Journal of Surgery*.

[15] Bansal V. K., Krishna A., Manek P. (2017). A Prospective Randomized Comparison of Testicular Functions, Sexual Functions and Quality of Life Following Laparoscopic Totally Extra-Peritoneal (TEP) and Trans-Abdominal Pre-Peritoneal (TAPP) Inguinal Hernia Repairs. *Surgical Endoscopy*.

[16] Campanelli G. (2023). Primary Inguinal Hernia, Postoperative Chronic Pain and Quality of Life. *Hernia*.

[17] Gupta S., Krishna A., Jain M. (2021). A Three-Arm Randomized Study to Compare Sexual Functions and Fertility Indices Following Open Mesh Hernioplasty (OMH), Laparoscopic Totally Extra Peritoneal (TEP) and Transabdominal Preperitoneal (TAPP) Repair of Groin Hernia. *Surgical Endoscopy*.

[18] Symeonidis E. N., Nasioudis D., Economopoulos K. P. (2016). Laparoendoscopic Single-Site Surgery (LESS) for Major Urological Procedures in the Pediatric Population: A Systematic Review. *International Journal of Surgery*.

